# Poor statistical power in population-based association study of gene interaction

**DOI:** 10.1186/s12920-024-01884-w

**Published:** 2024-04-27

**Authors:** Jiarui Ma, Jian Li, Yuqi Chen, Zhen Yang, Yungang He

**Affiliations:** 1 Shanghai Key Laboratory of Medical Epigenetics, International Co-Laboratory of Medical Epigenetics and Metabolism (Ministry of Science and Technology), Institutes of Biomedical Sciences, Fudan University, Shanghai, 200032 China; 2 Center for Medical Research and Innovation of Pudong Hospital, Intelligent Medicine Institute, Fudan University, Shanghai, 200032 China; 3 Shanghai Fifth People’s Hospital, Intelligent Medicine Institute, Fudan University, Shanghai, 200032 PR China

**Keywords:** Genetic association study, Gene interaction, Statistical epistasis, Statistical power

## Abstract

**Background:**

Statistical epistasis, or “gene–gene interaction” in genetic association studies, means the nonadditive effects between the polymorphic sites on two different genes affecting the same phenotype. In the genetic association analysis of complex traits, nevertheless, the researchers haven’t found enough clues of statistical epistasis so far.

**Methods:**

We developed a statistical model where the statistical epistasis was presented as an extra linkage disequilibrium between the polymorphic sites of different risk genes. The power of statistical test for identifying the gene–gene interaction was calculated and then compared in different hypothesis scenarios.

**Results:**

Our results show the statistical power increases with the increasing of interaction coefficient, relative risk, and linkage disequilibrium with genetic markers. However, the power of interaction discovery is much lower than that of regular single-site association test. When rigorous criteria were employed in statistical tests, the identification of gene–gene interaction became a very difficult task. Since the criterion of significance was given to be *p*-value ≤ 5.0 × 10^–8^, the same as that of many genome-wide association studies, there is little chance to identify the gene–gene interaction in all kind of circumstances.

**Conclusions:**

The lack of epistasis tends to be an inevitable result caused by the statistical principles of methods in the genetic association studies and therefore is the inherent characteristic of the research itself.

**Supplementary Information:**

The online version contains supplementary material available at 10.1186/s12920-024-01884-w.

## Background

Gene interaction, or epistasis, is an important concept in biochemical genetics, population genetics and quantitative genetics [1]. The specific definitions of gene interaction are different in different fields, but its basic concept is the interaction among genetic loci in their effects on phenotypes or fitness [2].

Gene interaction plays an important role in molecular genetics and quantitative genetics [1, 3]. The type of gene interaction determines the functional relationship between genes, genes and pathways, and the corresponding gene products [4, 5]. The degree and nature of gene interactions are also important for the theoretical explanation of the selective advantage of sexual reproduction [6, 7]. In addition, gene interaction has a profound effect on quantitative genetics since it plays an important role in many evolutionary processes, and may be involved in the reproductive isolation of species, the additive genetic variance caused by population bottlenecks, the evolution of trait mutation stability, adaptive gene complexes, sexual reproduction, and genetic system structures [3, 8].

Ronald A. Fisher explained the gene interaction from a statistical perspective for the first time and described gene interaction as a non-additive statistical effect between genetic polymorphic sites on two different genes which affect the same biological phenotype [9]. The modern quantitative genetics proposed by Cockerham et al. further developed the definition of gene interaction and regarded it as an interaction term in the regression of allelic effects [10]. The statistical gene interaction of two genes suggests that they also have physiological gene interaction, which may be caused by direct interactions between proteins or indirect interactions of gene product interaction networks. Therefore, statistical gene interaction can provide insights into the genetic structure of complex phenotypes, which may help to improve the statistical power of genetic association analysis.

In the past ten years, genome-wide genetic association studies (GWAS) have gradually emerged, and a large number of research results on genetic markers about polygenic phenotypes have also emerged in abundance. For example, 45 lung cancer susceptibility loci have been reported with different strength of evidence, highlighting suspected causal genes at each locus [11]. However, the influence of a single genetic marker is limited and cannot fully explain the corresponding phenotypic genetic variance. Therefore, investigators tried to combine multiple genetic markers for statistical analysis, hoping to enhance the statistical power of genetic association analysis through "gene interaction", and find the source of unexplained genetic variance and more undiscovered genetic markers and achieve scientific breakthroughs [12]. The first and only genome-wide two-locus interaction analysis about lung cancer performed so far by Minjie Chu et al. revealed a significant interaction between SNPs rs2562796 and rs16832404 with a small sample size (858 cases and 1115 controls). Since small sample size may easily result in false positives, further studies with large sample size are needed to validate their findings [13]. In large-scale genetic association analysis, gene interactions with statistical meaning have not yet been found.

Some investigators attributed this problem to the limitations of linear models. The linear model plays an important role in modern genetic epidemiology because of its solid theoretical foundation and widespread usage in different software packages [14]. Despite the advantages of using linear models, they do have limitations for explaining genetic models of disease due to limited ability to detect nonlinear patterns of genetic interaction [15]. The limitations of statistical models have promoted the development of calculation methods such as machine learning and data mining. The methods reviewed by Cordell [16] include novel approaches such as combinatorial partitioning [17], logic regression [18], and machine learning approaches such as random forests [19]. Based on logistic regression model, Cordell et al. carried out comprehensive analyses for detecting gene–gene interactions and obtained generally decent power under specific scenarios [20]. These novel methods, nevertheless, have limitations in their application. For example, multifactor dimensionality reduction (MDR), a significant new method for detecting and characterizing patterns of statistical epistasis in genetic association studies, was feasible only for examining two-locus interactions in a filtered data set or for examining higher-level interactions in an even further reduced data set [16]. GWAS poses greater challenges to computational methods. As summarized by Ritchie [21] and Moore [22], combinatorial assessment of SNPs in a GWAS is not computationally feasible beyond exploring two-way and three-way combinations. Analysis tools and software that can detect statistical gene interaction quickly and accurately are lacking yet [14].

Research on the detection of gene interaction in genetic association analysis is fruitful, and there are continuous innovations in technical means, but there are also shortcomings, that is, too much attention is paid to modeling and calculation methods, and little attention is paid to the fundamental question of whether gene interaction exists in genetic association analysis. To overcome these limitations, we established an additive genetic effect model with two alleles and developed a measure of interaction between two unlinked disease alleles under the framework of LD analysis to investigate whether there exists statistical epistasis in genetic association studies. We then calculated the power of statistical testing methods to detect gene interactions in different genetic scenarios. As a reference, we conducted a regular single-site genetic association study between the marker gene and the disease under different genetic scenarios, and obtained decent statistical power with reasonable parameters. For exploring the possible solution for the interaction identification, we evaluated the statistic power in investigation of interaction between two unlinked loci under parameters as same as the aforementioned single-site genetic association study and concluded with a reasonable theoretical explanation for the phenomenon of "missing gene interaction" in genetic association analysis.

## Methods

### Case-control model

To investigate the LD pattern generated by gene–gene interaction, we assume that the disease-susceptibility loci are in Hardy–Weinberg equilibrium (HWE) in the population. Let X_1_ and X_2_ be the two independent disease genes located on the same chromosome. We give $P(X_{1}|D)$ and $P(X_{2}|D)$ as the probabilities of carrying the disease gene X_1_ and X_2_ by the affected individual, respectively. When $P(X_{1},X_{2}|D)$ is the probability of carrying both X_1_ and X_2_, the LD between X_1_ and X_2_ in the affected individuals due to their interaction can be obtained in $D = P(X_{1},X_{2}|D) - P(X_{1}|D) \cdot P(X_{2}|D)$.

When there is a gene–gene interaction between X_1_ and X_2_, the coefficient of interaction could be defined in the disease case–control study as $K = \frac{{f_{11D} }}{{f_{1*D}f_{*1D} }} - 1$, where $f_{1*D}$, $f_{*1D}$, and $f_{11D}$ is frequency of chromosomes in affected individuals that carrying X_1_, X_2_, and both of them, respectively. The notation ‘*’ indicates any allele of ‘0’ or ‘1’ at the current site, i.e. $f_{1*D} = f_{11D} + f_{10D}$.We assume that the relative risks of X_1_ and X_2_ are *R*_*X1*_ and *R*_*X2*_, respectively. We follow the definition of relative risks in the literature [23]. *R*_*X1*_ is the ratio of the incidence of individuals carrying X_1_ in the population to the incidence of individuals without X_1_. In the same way, we can define *R*_*X2*_. The aforementioned frequencies in affected individuals are assumed as $f_{1*D} = \frac{{R_{X1} f_{1*} }}{{R_{X1} f_{1*} + (1 - f_{1*} )}}$, $f_{*1D} = \frac{{R_{X2} f_{*1} }}{{R_{X2} f_{*1} + (1 - f_{*1} )}}$, and $f_{11D} { = (1 + }K{)} \cdot f_{1*D} f_{*1D}$, where $f_{1*}$ and $f_{*1}$ is frequency of chromosomes in the population that carrying X_1_, X_2_, respectively. It is clear that the interaction coefficient K indicates the extra LD between X_1_ and X_2_ due to their non-additive (multiplicative) risk. Similarly, we can obtain the frequencies $f_{1*d}$, $f_{*1d}$, and $f_{11d}$ for the chromosomes in unaffected individuals.

When the disease-susceptibility loci are usually undetectable, genetic association studies are employed with marker genes. In this study, we assume that the marker gene M_1_ is in LD with X_1_ and that the marker gene M_2_ is in LD with X_2_. Furthermore, we assume that X_1_ and M_2_ are unlinked and that X_2_ and M_1_ are unlinked.

### Parameters of the model

For the case–control study, we have N_D_ chromosomes in the affected group and N_d_ chromosomes in the control group. According to their carrying alleles of the M_1_ and M_2_ genes, the affected group and control group can be further categorized into more subgroups (Table 1). For example, the affected group with alleles ‘1’ of M_1_ and ‘0’ of M_2_ have a size of N_10D_; the control group with alleles ‘0’ of M_1_ and ‘1’ of M_2_ have a size of N_01d_.

**Table 1:** Grouping table of disease population and general population on M_1_ and M_2_ Alleles

Markers	Affected group	Control population
M_1_ = 0, M_2_ = 0	N_00D_	N_00d_
M_1_ = 0, M_2_ = 1	N_01D_	N_01d_
M_1_ = 1, M_2_ = 0	N_10D_	N_10d_
M_1_ = 1, M_2_ = 1	N_11D_	N_11d_
Total size	N_D_	N_d_

The numbers of chromosomes carrying different genetic markers follow a multinomial distribution with the given sample sizes (*N*_*D*_ and *N*_*d*_) and frequency vector, i.e. $[p_{00D} ,p_{01D} ,p_{10D} ,p_{11D} ]^{T}$ and $[p_{00d} ,p_{01d} ,p_{10d} ,p_{11d} ]^{T}$. For simplicity, in this study, we assumed the cases and controls have the same size, i.e. *N*_*D*_ = *N*_*d*_. The frequencies of different chromosomes in affected or unaffected individuals can be obtained with the disease prevalence, the relative risks of disease alleles, the interaction coefficient, LDs and allele frequencies of suspect, and marker genes in the general population. For example, the expected frequency of chromosomes carrying allele ‘1’ at both M_1_ and M_2_ genes can be calculated for the affected individuals as below.

$$\begin{gathered} p_{11D} = \frac{{(p_{1*} f_{1*} + D_{X1,M1} )(p_{*1} f_{*1} + D_{X2,M2} )f_{11D} }}{{f_{1*} f_{*1} }} + \\ \frac{{[p_{1*} (1 - f_{1*} ) + D_{X1,M1} ](p_{*1} f_{*1} + D_{X2,M2} )f_{01D} }}{{(1 - f_{1*} )f_{*1} }} + \\ \frac{{(p_{1*} f_{1*} + D_{X1,M1} )[p_{*1} (1 - f_{*1} ) + D_{X2,M2} ]f_{10D} }}{{f_{1*} (1 - f_{*1} )}} + \\ \frac{{[p_{1*} (1 - f_{1*} ) + D_{X1,M1} ][p_{*1} (1 - f_{*1} ) + D_{X2,M2} ]f_{00D} }}{{(1 - f_{1*} )(1 - f_{*1} )}} \\ \end{gathered}$$

where the notation *p* indicates the frequencies of genetic markers (M_1_ and M_2_) and the notation *f* notes the frequencies of disease genes (X_1_ and X_2_). LD between the gene makers and disease genes is presented in *D*. Assume that *D*_*1*_*’* is the Lewontin's linkage disequilibrium value between X_1_ and M_1_ and *D*_*2*_*’* is the Lewontin's linkage disequilibrium value between X_2_ and M_2_. More details of the calculation for different groups can be found in the Additional file 1.

### Expectation and variance of effect size

We cited the LD-based definition of genetic interaction of Zhao et al. [12] in this research. The LD between the marker genes M_1_ and M_2_ for the affected and unaffected individuals are defined as $\beta_{D} = p_{11D} - p_{1*D} p_{*1D}$ and $\beta_{d} = p_{11d} - p_{1*d} p_{*1d}$, respectively. Then $\hat{\beta } = \hat{\beta }_{D} - \hat{\beta }_{d}$ is the estimated effect size of gene–gene interaction in a case–control study.

As the case and control samples are collected independently, the expectation of estimated effect size can be calculated as $E(\hat{\beta }) = E(\hat{\beta }_{D} ) - E(\hat{\beta }_{d} )$. It is easy to obtain expectations.

$$\begin{gathered} E(\hat{\beta }_{D} ) = {p_{11D}} p_{00D} - p_{10D} p_{01D} - Cov(\hat{p}_{1*D} ,\hat{p}_{*1D} ) \hfill \\ E(\hat{\beta }_{d} ) = p_{11d} p_{00d} - p_{10d} p_{01d} - Cov(\hat{p}_{1*d} ,\hat{p}_{*1d} ) \hfill \\ \end{gathered}$$

The covariance between $\hat{p}_{1*D}$ and $\hat{p}_{*1D}$ are defined as $Cov(\hat{p}_{1*D} ,\hat{p}_{*1D} )$, and the covariance between $\hat{p}_{1*d}$ and $\hat{p}_{*1d}$ are defined as $Cov(\hat{p}_{1*d} ,\hat{p}_{*1d} )$, which can be calculated as.

$$\begin{gathered} Cov(\hat{p}_{1*D} ,\hat{p}_{*1D} ) = \frac{{p_{11D} - p_{1*D} p_{*1D} }}{{N_{D} }} \hfill \\ Cov(\hat{p}_{1*d} ,\hat{p}_{*1d} ) = \frac{{p_{11d} - p_{1*d} p_{*1d} }}{{N_{d} }} \hfill \\ \end{gathered}$$

More details could be found in the Additional file 1.

As both the sample size N_D_ and N_d_ are large enough, the above mentioned covariance is ignorable. We therefore calculate the expectation of effect size as of below.

$$E(\hat{\beta }) = (p_{11D} p_{00D} - p_{10D} p_{01D} ) - (p_{11d} p_{00d} - p_{10d} p_{01d} )$$

We have $Var(\hat{\beta }) = Var(\hat{\beta }_{D} ) + Var(\hat{\beta }_{d} )$. As the calculation of variance is complicated, we cited the results from the reference [12] directly as below.

$$\begin{gathered} Var(\hat{\beta }_{D} ) = \frac{1}{{N_{D} }}\left[ \begin{gathered} (p_{11D} + p_{10D} )(1 - p_{11D} - p_{10D} )(p_{11D} + p_{01D} )(1 - p_{11D} - p_{01D} ) + \hfill \\ (1 - 2p_{11D} - 2p_{10D} )(1 - 2p_{11D} - 2p_{01D} ) \cdot (p_{11D} p_{00D} - p_{10D} p_{01D} ) - \hfill \\ (p_{11D} p_{00D} - p_{10D} p_{01D} )^{2} \hfill \\ \end{gathered} \right] \hfill \\ Var(\hat{\beta }_{d} ) = \frac{1}{{N_{d} }}\left[ \begin{gathered} (p_{11d} + p_{10d} )(1 - p_{11d} - p_{10d} )(p_{11d} + p_{01d} )(1 - p_{11d} - p_{01d} ) + \hfill \\ (1 - 2p_{11d} - 2p_{10d} )(1 - 2p_{11d} - 2p_{01d} )(p_{11d} p_{00d} - p_{10d} p_{01d} ) - \hfill \\ (p_{11d} p_{00d} - p_{10d} p_{01d} )^{2} \hfill \\ \end{gathered} \right] \hfill \\ \end{gathered}$$

### Statistical test and power calculation

We propose the statistic as below.

$$S = \frac{{\hat{\beta }^{2} }}{{Var(\hat{\beta })}}$$

when the distribution of effect size can be approximated in a normal distribution. We gave the null hypothesis (H_0_) $\beta = 0$ that there is no interaction between X_1_ and X_2_ and the alternative hypothesis (H_1_) $\beta \ne 0$, that is, there exists an interaction between X_1_ and X_2_. Under the null hypothesis, the statistic S follows a central chi-square distribution with a degree of freedom 1. The statistical power of the hypothesis testing can be obtained for different scenarios with different parameters. Details of the statistical test and power calculation were given in the Additional file 1.

## Results

### Expectation and variance of the effect size

Effect size presents the magnitude of differences found in the genetic analysis of various scenarios. To investigate how the gene–gene interaction coefficient *K* and relative risk *R*_*x*_ (*R*_*X1*_ and *R*_*X2*_) affect the effect size, we calculated expectation of effect size $E(\beta )$ with varying *K* and *R*_*x*_. In this evaluation, we keep other parameters constant. Assume that the X_1_ and M_1_, X_2_ and M_2_ are fully linked (i.e. *D*_*1*_*’* = 1, *D*_*2*_*’* = 1). The risk allele frequencies of all genes (X_1_, X_2_, M_1_, and M_2_) are assigned to be 0.05 when the disease prevalence is given as 0.1.

**Fig. 1:**
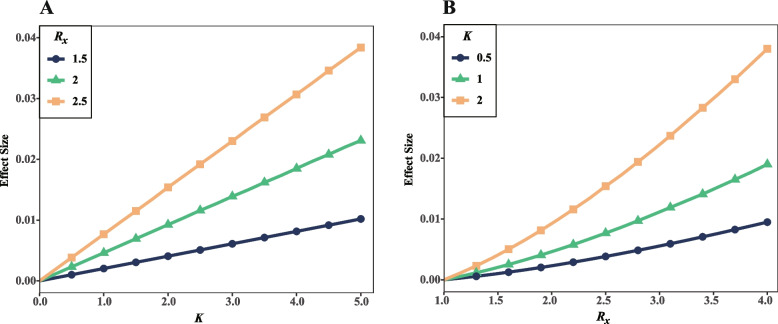
Interaction coefficient and relative risks contribute to effect size in case–control studies. **A** The relationships between $E(\beta )$ and *K* with different *R*_*x*_. **B** The relationship between $E(\beta )$ and *R*_*x*_ under different *K*

Our results show the expectation of effect size linearly inflate with the increasing of interaction when relative risks of both disease genes are 1.5, 2.0, and 2.5, respectively (Fig. 1A). When the interaction coefficient is only 1.0, the effect sizes are 2.04 × 10^–3^, 4.62 × 10^–3^, and 7.68 × 10^–3^, respectively. However, the effect sizes increase to 4.07 × 10^–3^, 9.25 × 10^–3^, and 1.54 × 10^–2^ when the interaction coefficient was improved to 2.0. The higher the risk, the greater the increasing rate of effect size.

Being different from the aforementioned linear relationship, the effect size shows an exponential increase with higher relative risk (Fig. 1B). The effect sizes are 1.02 × 10^–3^, 2.04 × 10^–3^ and 4.07 × 10^–3^ for the interaction of 0.5, 1.0, and 2.0 when the relative risks of both disease genes are given as 1.5. Alternatively, with the twofold higher relative risks (*R*_*x*_ = 3.0), the effect sizes become 5.56 × 10^–3^, 1.11 × 10^–2^, and 2.23 × 10^–2^, respectively. It indicates a nonlinear relationship of impact factors.

**Fig. 2:**
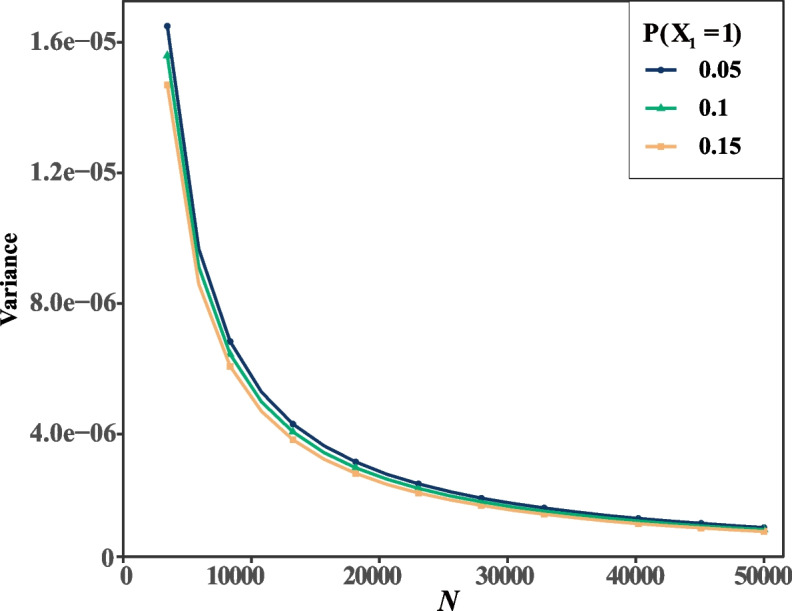
Sample sizes contribute to variance in case–control studies

It is critical to accurately estimate effect size in the genetic analysis. We therefore calculated the variance with varying sample sizes to discover their relationship. In this study, we give the interaction coefficient *K* = 1.0 and relative risks of both disease genes* R*_*x*_ = 2.0 when the other parameters are the same as that of before. Our results show the variance of effect size rapidly decline with the increasing of sample size when the risk allele frequencies of X_1_ are 0.05, 0.1, and 0.15, respectively (Fig. 2). With the sample size *N* = *N*_*D*_ = *N*_*d*_ = 5000, the variances are 1.14 × 10^–5^, 1.12 × 10^–5^, and 1.09 × 10^–5^, respectively. When the size are the twofold large (*N* = 10000), the variances become 5.71 × 10^–6^, 5.58 × 10^–6^, and 5.45 × 10^–6^, respectively. It indicates a nonlinear relationship between the variance and sample size.

### Statistical power of a single test

**Fig. 3:**
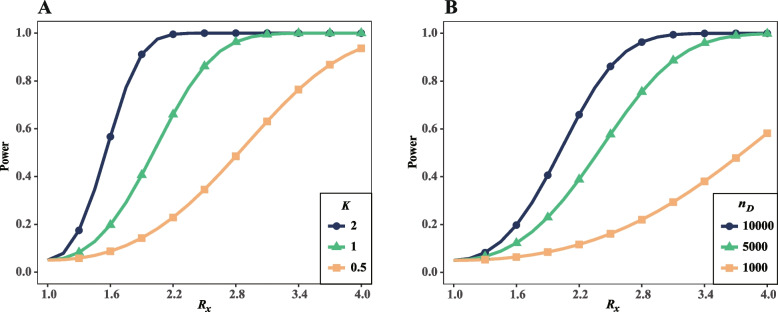
Relative risks contribute to power in case–control studies. **A** The relationships between power and *R*_*x*_ with different* K*. **B** The relationship between power and *R*_*x*_ under different sample sizes

Statistical power is the central issue for the discovery of gene–gene interactions in genetic association studies. To investigate how the relative risk *R*_*x*_ contributed the power, we calculated the statistical power with varying *R*_*x*_ and the significant criterion *p*-value ≤ 0.05. The other parameters are the same as that of before if there lacks further statement. Our results show the substantial increase of statistical power with the inflating of relative risk *R*_*x*_ and the interaction coefficients *K* under the circumstance where the sample sizes of both case and control groups are 10,000 chromosomes (Fig. 3A). Since the interaction coefficient is as large as 2.0, the statistical power is 0.10 with *R*_*x*_ = 1.20 and 0.83 with *R*_*x*_ = 1.80. The statistical power is significantly weak when the interaction coefficient was setback to 1.0 or 0.5. Specifically, in the scenario with interaction coefficient 0.5, the power is only 0.05 for *R*_*x*_ = 1.20 and 0.12 for *R*_*x*_ = 1.80, respectively. Because relative risk of disease gene is generally weak, the aforementioned results indicate the gene–gene interaction is difficult to identify in such circumstances. As sample size could be different in different studies, we further evaluate the power in scenarios with varying sample sizes and the interaction coefficient *K* = 1. When the sample sizes are 1000, 5000, and 10,000, the statistical power are 0.09, 0.28, and 0.49 with relative risk *R*_*x*_ = 2.0, respectively (Fig. 3B). The results show a great sample size is necessary for identifying gene–gene interaction even if the relative risks of disease genes are enormously large.

**Fig. 4:**
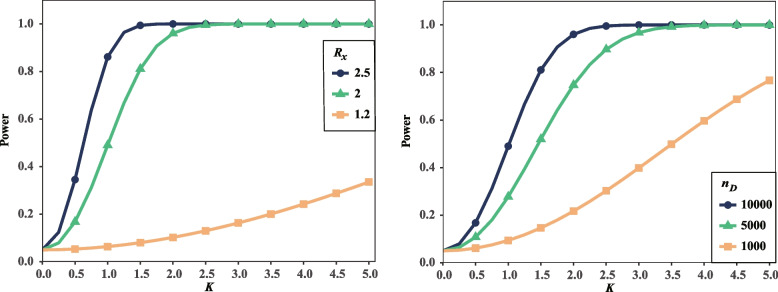
Interaction coefficient contributes to power in case–control studies. **A** The relationships between power and *K* with different *R*_*x*_. **B** The relationship between power and *K* under different sample sizes

To investigate how the interaction coefficient *K* affect the statistical power, we calculated power with varying *K*. In this evaluation, we keep other parameters the same as before. Our results show the power nonlinearly inflate with the increasing of interaction coefficient (Fig. 4A). When the interaction coefficient is weak (*K* = 1.0), the power is only 0.06 with *R*_*x*_ = 1.2, 0.49 with *R*_*x*_ = 2.0, and 0.86 with *R*_*x*_ = 2.5, respectively. Alternatively, the power increases to 0.10, 0.96, and 0.99 when the interaction coefficient was improved to *K* = 2.0. Our results show the power approximate to 1.0 in the circumstances with the large relative risks and strong interactions. However, the circumstances could be rare in genetic association studies of real world.

We also investigated the statistical power with both varying interaction coefficient and different sample sizes (Fig. 4B). As that was expected, the power is weak when the sample sizes are limited. With the sample size being 1000 and *R*_*x*_ = 2.0, the power is as small as 0.09 for *K* = 1.0, 0.22 for *K* = 2.0, respectively.

### Statistical power with multiple testing corrections

Genetic association studies usually involved tens of thousands of genetic markers. It is therefore important to evaluate the statistic power for identification of gene–gene interaction in such stricter criterion in statistical tests. To explore the possible solution for the interaction identification, we evaluated the statistic power with different LD and allele frequencies under varying significant criteria.

By assuming that the X_1_ and M_1_ are not fully linked but X_2_ and M_2_ are in complete LD (i.e. *D*_*1*_*’* ≠ 1, *D*_*2*_*’* = 1). We investigate how *D*_*1*_*’* affects the statistical power under different levels of significance. In this evaluation, we keep other parameters constant. Among them, the risk allele frequencies of X_2_, M_1_, and M_2_ are assigned to be 0.2 when the sample size are 10,000. We also have *K* = 1.0 and *R*_*x*_ = 2.0 with the disease prevalence 0.1.

**Fig. 5:**
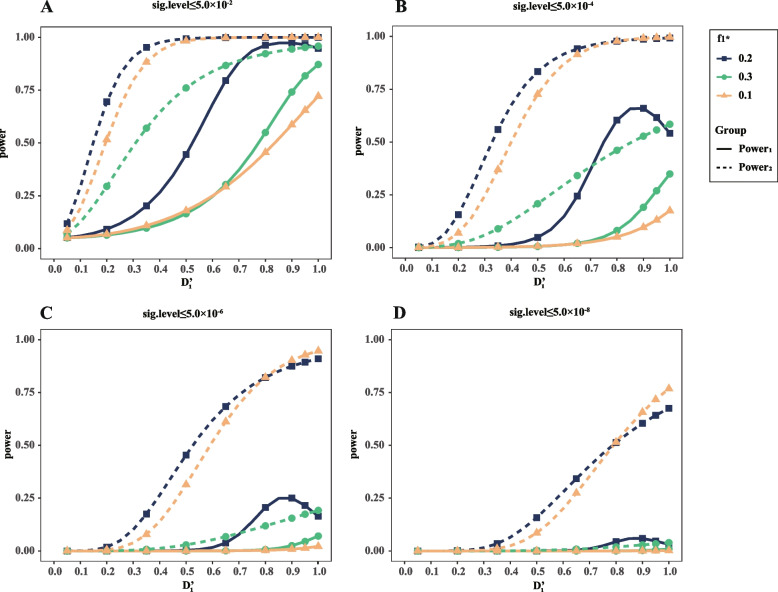
*D*_*1*_*’* and level of significance contributes to power_1_ and power_2_. **A-D** The figures show the relationship between power_1_, power_2_, and *D*_*1*_*’* under three groups of $f_{1*}$ when level of significance ≤ 5.0 × 10^–2^, ≤ 5.0 × 10^–4^, ≤ 5.0 × 10^–6^, and ≤ 5.0 × 10^–8^, respectively

Our results show the statistical power generally increases with the increasing of *D*_*1*_*’* (Fig. 5A, Power_1_). When the LD is moderate, say *D*_*1*_*’* = 0.5, the power is only 0.18, 0.45, and 0.16 for the risk allele frequency of X_1_ being 0.1, 0.2, and 0.3. Alternatively, with *D*_*1*_*’* = 1.0, the power was improved to 0.72, 0.95, and 0.87 for the different allele frequencies, respectively. To compare the aforementioned power of interaction identification with that of regular association test, we calculated the power of association test for the marker gene M_1_ in the same circumstances. Results show the power of regular association test is much higher than that of interaction discovery (Fig. 5A, Power_2_). When much stricter criteria (*sig.level* ≤ 5.0 × 10^–4^, ≤ 5.0 × 10^–6^, and ≤ 5.0 × 10^–8^) were applied in our investigation, the identification of gene–gene interaction became more difficult than ever before (Fig. 5B, C, D). As the significance level was given the same as many genome-wide association studies, *p*-value ≤ 5.0 × 10^–8^, there is little opportunity to identify the gene–gene interaction in all kind of circumstances (Fig. 5D). However, as that was expected, it still possible to discover the significant association between the marker gene M_1_ and the disease (Fig. 5D).

**Fig. 6:**
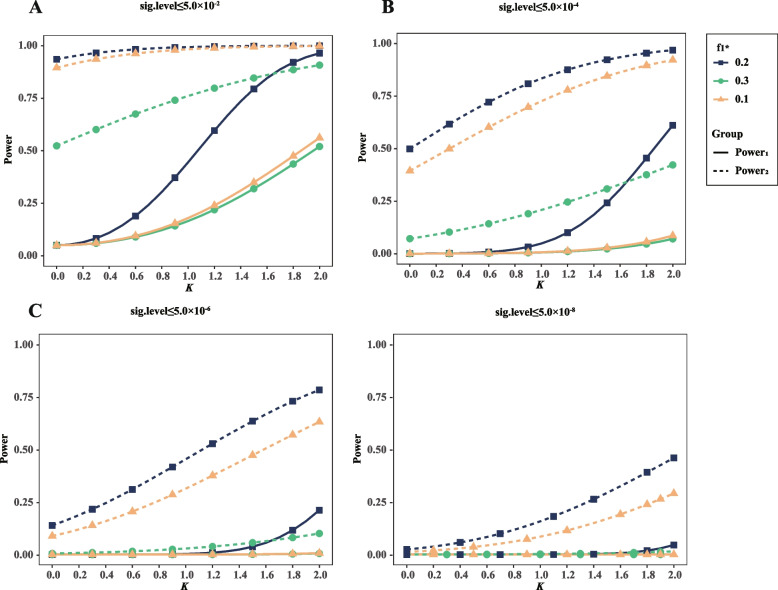
The interaction coefficient and level of significance contributes to power_1_ and power_2_. **A-D** The figures show the relationship between power_1_, power_2_, and *K* under three groups of $f_{1*}$ when level of significance ≤ 5.0 × 10^–2^, ≤ 5.0 × 10^–4^, ≤ 5.0 × 10^–6^, and ≤ 5.0 × 10^–8^, respectively

To investigate the effects of interaction coefficient *K*, both the power of interaction identification and the power of regular genetic association test were obtained for different interaction coefficients and different significance levels. Here we gave *D*_*1*_*’* and *D*_*2*_*’* to be 0.5 and 1.0 respectively, when other parameters were the same as that aforementioned. Our results show the statistical power of both of the tests increase with the increasing of *K* in general scenarios (Fig. 6). However, with the criterion *p*-value ≤ 5.0 × 10^–8^, it is little of power to identify the gene–gene interaction even if the interaction coefficient is as large as 2.0 (Fig. 6D).

## Discussion

Because the influence of a single genetic variant is limited, in order to find the source of unexplained genetic variance and more undiscovered genetic markers, investigators tried to combine multiple genetic markers in their analysis to enhance the statistical power of genetic association study through "gene–gene interaction". Wu and Cui proposed a varying-coefficient model for the detection of nonlinear gene-environment interaction with binary disease traits and got decent power in a variety of genetic settings given reasonable control on false positive rate [24]. However, no similar results have been found in the studies of gene–gene interaction, and the reasons of which have aroused many debates. Some investigators argued that this problem is caused by technical limitations, and a large variety of regularization models for interaction studies have been developed, including regression based, LD based, Bayesian, data filtering and machine learning methods, etc [25, 26]. Nevertheless, the progress of statistical theory and calculation methods have not yet brought substantial help to solve the problem. Our study provided an alternative perspective to the long debates that the gene–gene interaction may not be detectable due to the lack of statistical power.

A large number of genetic markers in actual genetic association studies make it difficult to obtain decent statistical power. For controlling false positive results, multiple test corrections are required to be conducted. Taking the Bonferroni method as an example, for a multiple test with around 1.0 × 10^6^ genetic markers, the declared significant threshold after Bonferroni correction is about 5.0 × 10^–8^. Our results showed that the power of testing gene–gene interaction is close to zero under such rigorous threshold. Kraft et al. developed a joint test of marginal associations and showed that the joint test could have higher power than other joint tests for detecting the gene-environment association [27]. Their method is also helpful to detect the gene–gene interaction due to similar forms of statistical models of the gene-environment and gene–gene interactions. However, as the size of hypotheses for detecting gene–gene interactions is a quadratic function of marker numbers, the chance of finding statistical epistasis will be even lower in the genome-wide analysis with much rigorous significance criterion.

In genetic association studies, it is particularly important to rationally define and measure gene interactions. However, there is no consensus on what values of interaction parameter or epistatic models are appropriate for human disease and investigators have divergent mathematical definition of gene interaction strength. In the study of Gauderman [28, 29], gene interaction strength was related with the interaction regression coefficient of logistic model, while Zhao [12] defined it as a measure of interaction between two unlinked loci associated with the penetrance of different genotypes at the disease-susceptibility locus. In this study, we define coefficient of gene interaction as a concise ratio of allele frequencies about two independent disease genes located on the same chromosome, and the magnitude of which reflects the strength of the interaction, thus providing a foundation for developing statistics for detection of interaction.

An important benefit from investigating the genetics of human disease is to predict the risk that individuals may have of succumbing to a specific disease, the knowledge of which can then be used by the clinician in prevention, diagnosis, prognosis, and treatment [30]. That is why the relative risk is an important parameter in the studies of gene–gene interaction and it is significant to use reasonable value of relative risk when conducting theoretical research. The relative risk of disease due to one allele is typically of the order of 1.1 to 2.0 in the real world [30, 31], and the values in this range has also been used in previous studies of gene–gene interaction [12]. In this study, we evaluated the statistic power with different LD, allele frequencies and interaction coefficient under reasonable relative risks, ensuring the reliability of the results.

We selected the haplotype model as the research model for highlighting the main results. In the reference Hu et al. [32], the authors mentioned three different definitions of genetic interaction as the penetrance-based definition, the logit-based definition, and the LD-based definition. They showed that the 3 definitions are equivalent under the circumstances that linkage equilibrium holds in general population for the two loci dominant (or recessive) disease model. Since the framework of their genotype model researching gene–gene interaction applies as well in haplotype model when haplotype at two loci can be inferred from diplotype without uncertainty [32], our conclusions about gene–gene interaction apply to dominant and recessive inheritance patterns under Hardy–Weinberg equilibrium.

The aforementioned report [32] discussed the power of statistical tests to identify genetic interactions and emphasized the significance of the power in the specific circumstances. In our study, we assumed that the casual variants are not determined but in LD with the detected markers. Alternatively, the previous report gave the effect sizes of disease risk and interaction to the detected markers. The previous report may overestimate the power in general circumstances because there usually lacks a complete association between causal variants and genetic markers. The primary difference explained the different results in our research and the previous studies.

The results in this article are preliminary. Interaction between high-order interactions among multiple loci have not been studied. Further research is required to investigate gene–gene interactions among multiple loci and explore the problem of missing statistical epistasis in genetic association analysis in a more in-depth and comprehensive manner, so as to lay a foundation for the optimization of genetic association analysis methods. In addition, this analysis is not applicable to general disease models with a total of nine genotypes since our research is based on haplotype model. For more general disease models, dominant coding or recessive coding for each of two loci [29, 32] can be employed to study gene–gene interactions as done in the previous reports.

## Conclusions

This study provides a possible explanation for the long-standing important question of "lack of epistasis" in genetic association studies. It points out that the lack of epistasis tends to be an inevitable result caused by the statistical principles of genetic association research methods and is the inherent characteristic of the research method itself. The results of this study suggest that looking for epistasis from genetic association studies is an extremely tough task.

### Supplementary Information

**Additional file 1.** Supplementary Methods.

## Data Availability

The data used in this study are all simulated data. The related R program can be obtained from Github at jrma21/Poor-Statistical-Power-in-Population-based-Association-Study-of-Gene-Interaction (github.com).
