# Supplementary material for: Poor statistical power in population-based association study of gene interaction
Source: BMC Med Genomics. 2024 Apr 27;17:111. doi: 10.1186/s12920-024-01884-w (PMC11055307; doi:10.1186/s12920-024-01884-w)
Supplement: Supplementary file 1 — Additional file 1. Supplementary Methods. [file 12920_2024_1884_MOESM1_ESM.pdf]

Supplementary Methods

1. The calculation process of $p_{11D}, p_{10D}, p_{00D}, p_{01D}, p_{11d}, p_{10d}, p_{00d}$ and p_{01d}

As X1, X2, M1 and M2 have two possible values: 0 and 1 respectively, p_{11D} can be calculated using Multiplicative Theorem of Conditional Probability as below

$$
\begin{aligned}
&P_{11D} = P(M_1=1, M_2=1, X_1=1, X_2=1 \mid D) + P(M_1=1, M_2=1, X_1=0, X_2=1 \mid D) + \\
&P(M_1=1, M_2=1, X_1=1, X_2=0 \mid D) + P(M_1=1, M_2=1, X_1=0, X_2=0 \mid D) \\
&= P(M_1=1, M_2=1 \mid X_1=1, X_2=1) \cdot P(X_1=1, X_2=1 \mid D) + \\
&P(M_1=1, M_2=1 \mid X_1=0, X_2=1) \cdot P(X_1=0, X_2=1 \mid D) + \\
&P(M_1=1, M_2=1 \mid X_1=1, X_2=0) \cdot P(X_1=1, X_2=0 \mid D) + \\
&P(M_1=1, M_2=1 \mid X_1=0, X_2=0) \cdot P(X_1=0, X_2=0 \mid D)
\end{aligned}
.
$$

Because X1 and M2 are unlinked and X2 and M1 are unlinked, we can conclude that

$$
\begin{aligned}
&P(M_1=1, M_2=1 \mid X_1=1, X_2=1) = P(M_1=1 \mid X_1=1) \cdot P(M_2=1 \mid X_2=1) \\
&P(M_1=1, M_2=1 \mid X_1=0, X_2=1) = P(M_1=1 \mid X_1=0) \cdot P(M_2=1 \mid X_2=1) \\
&P(M_1=1, M_2=1 \mid X_1=1, X_2=0) = P(M_1=1 \mid X_1=1) \cdot P(M_2=1 \mid X_2=0) \\
&P(M_1=1, M_2=1 \mid X_1=0, X_2=0) = P(M_1=1 \mid X_1=0) \cdot P(M_2=1 \mid X_2=0)
\end{aligned}
.
$$

According to Bayesian conditional probability formula in the main body, eight conditional probabilities can be calculated as

$$
\begin{aligned}
P(M_1=1 \mid X_1=1) &= \frac{P(M_1=1, X_1=1)}{P(X_1=1)} = \frac{p_{1*} f_{1*} + D_{X1,M1}}{f_{1*}} \\
P(M_2=1 \mid X_2=1) &= \frac{P(M_2=1, X_2=1)}{P(X_2=1)} = \frac{p_{*1} f_{*1} + D_{X2,M2}}{f_{*1}} \\
P(M_1=1 \mid X_1=0) &= \frac{P(M_1=1, X_1=0)}{P(X_1=0)} = \frac{p_{1*} \cdot (1-f_{1*}) + D_{X1,M1}}{1-f_{1*}} \\
P(M_2=1 \mid X_2=0) &= \frac{P(M_2=1, X_2=0)}{P(X_2=0)} = \frac{p_{*1} \cdot (1-f_{*1}) + D_{X2,M2}}{1-f_{*1}} \\
P(M_1=0 \mid X_1=1) &= \frac{P(M_1=0, X_1=1)}{P(X_1=1)} = \frac{(1-p_{1*}) \cdot f_{1*} + D_{X1,M1}}{f_{1*}} \\
P(M_2=0 \mid X_2=1) &= \frac{P(M_2=0, X_2=1)}{P(X_2=1)} = \frac{(1-p_{*1}) \cdot f_{*1} + D_{X2,M2}}{f_{*1}} \\
P(M_1=0 \mid X_1=0) &= \frac{P(M_1=0, X_1=0)}{P(X_1=0)} = \frac{(1-p_{1*}) \cdot (1-f_{1*}) + D_{X1,M1}}{1-f_{1*}} \\
P(M_2=0 \mid X_2=0) &= \frac{P(M_2=0, X_2=0)}{P(X_2=0)} = \frac{(1-p_{*1}) \cdot (1-f_{*1}) + D_{X2,M2}}{1-f_{*1}}
\end{aligned}
.
$$

From the main body of the literature, we can get f_{11D}=$(1+K)\cdot f_{1*D}f_{*1D}$, and $f_{1*D}=f_{11D}+f_{10D}$, so we can conclude that

$$\begin{aligned}
f_{10D} &= f_{1*D}-f_{11D}=f_{1*D}-(1+K)\cdot f_{1*D}f_{*1D}\\
f_{01D} &= f_{*1D}-f_{11D}=f_{*1D}-(1+K)\cdot f_{1*D}f_{*1D}\\
f_{00D} &= 1-f_{11D}-f_{10D}-f_{01D}=1-f_{1*D}-f_{*1D}+(1+K)\cdot f_{1*D}f_{*1D}
\end{aligned}.$$

From the main body of the literature, we can get $f_{1*D}=\dfrac{R_{X1}f_{1*}}{R_{X1}f_{1*}+(1-f_{1*})}$, $f_{1*D}=\dfrac{R_{X2}f_{*1}}{R_{X2}f_{*1}+(1-f_{*1})}$, so the frequency of chromosomes carrying allele ‘1’ at both M_1 and M_2 genes in affected individuals can be calculated as below

$$\begin{aligned}
p_{11D}=&\frac{(p_{1*}f_{1*}+D_{X1,M1})(p_{*1}f_{*1}+D_{X2,M2})f_{11D}}{f_{1*}f_{*1}}+\ldots\\
&\frac{[p_{1*}(1-f_{1*})+D_{X1,M1}](p_{*1}f_{*1}+D_{X2,M2})f_{01D}}{(1-f_{1*})f_{*1}}+\ldots\\
&\frac{(p_{1*}f_{1*}+D_{X1,M1})[p_{*1}(1-f_{*1})+D_{X2,M2}]f_{10D}}{f_{1*}(1-f_{*1})}+\ldots\\
&\frac{[p_{1*}(1-f_{1*})+D_{X1,M1}][p_{*1}(1-f_{*1})+D_{X2,M2}]f_{00D}}{(1-f_{1*})(1-f_{*1})}
\end{aligned}.$$

In the same way, the frequency of chromosomes carrying allele ‘0’ at both M_1 and M_2 genes in affected individuals can be calculated as

$$\begin{aligned}
p_{00D}=&\frac{[(1-p_{1*})f_{1*}+D_{X1,M1}][(1-p_{*1})f_{*1}+D_{X2,M2}]f_{11D}}{f_{1*}f_{*1}}+\\
&\frac{[(1-p_{1*})(1-f_{1*})+D_{X1,M1}][(1-p_{*1})f_{*1}+D_{X2,M2}]f_{01D}}{(1-f_{1*})f_{*1}}+\\
&\frac{[(1-p_{1*})f_{1*}+D_{X1,M1}][(1-p_{*1})(1-f_{*1})+D_{X2,M2}]f_{10D}}{f_{1*}(1-f_{*1})}+\\
&\frac{[(1-p_{1*})(1-f_{1*})+D_{X1,M1}][(1-p_{*1})(1-f_{*1})+D_{X2,M2}]f_{00D}}{(1-f_{1*})(1-f_{*1})}
\end{aligned}.$$

The frequency of chromosomes carrying allele '1' at M_1, and allele '0' at M_2 in affected individuals can be calculated as

$$p_{10D} = \frac{(p_{1*}f_{1*} + D_{X1,M1})[(1-p_{*1})f_{*1} + D_{X2,M2}]f_{11D}}{f_{1*}f_{*1}} + \frac{[p_{1*}(1-f_{1*}) + D_{X1,M1}][(1-p_{*1})f_{*1} + D_{X2,M2}]f_{01D}}{(1-f_{1*})f_{*1}} + \frac{(p_{1*}f_{1*} + D_{X1,M1})[(1-p_{*1})(1-f_{*1}) + D_{X2,M2}]f_{10D}}{f_{1*}(1-f_{*1})} + \frac{[p_{1*}(1-f_{1*}) + D_{X1,M1}][(1-p_{*1})(1-f_{*1}) + D_{X2,M2}]f_{00D}}{(1-f_{1*})(1-f_{*1})}.$$

The frequency of chromosomes carrying allele '0' at M_1, and allele '1' at M_2 in affected individuals can be calculated as

$$p_{01D} = \frac{[(1-p_{1*})f_{1*} + D_{X1,M1}](p_{*1}f_{*1} + D_{X2,M2})f_{11D}}{f_{1*}f_{*1}} + \frac{[(1-p_{1*})(1-f_{1*}) + D_{X1,M1}](p_{*1}f_{*1} + D_{X2,M2})f_{01D}}{(1-f_{1*})f_{*1}} + \frac{[(1-p_{1*})f_{1*} + D_{X1,M1}][p_{*1}(1-f_{*1}) + D_{X2,M2}]f_{10D}}{f_{1*}(1-f_{*1})} + \frac{[(1-p_{1*})(1-f_{1*}) + D_{X1,M1}][p_{*1}(1-f_{*1}) + D_{X2,M2}]f_{00D}}{(1-f_{1*})(1-f_{*1})}.$$

In the unaffected individuals, the frequencies of chromosomes carrying specific allele at M_1 and M_2 genes are defined as p_{11d}, p_{10d}, p_{00d} and p_{01d} similarly. Assume that P_d is the disease prevalence, in order to calculate p_{11d}, p_{10d}, p_{00d} and p_{01d}, we assume relevant parameters j_a, j_b, j_c, j_d, j_1, and j_2 as follows

$$j_a = 1 - \frac{f_{1*D} \cdot p_d}{f_{1*}}, \quad j_b = 1 - \frac{(1-f_{1*D}) \cdot p_d}{1-f_{1*}},$$

$$j_c = 1 - \frac{f_{*1D} \cdot p_d}{f_{*1}}, \quad j_d = 1 - \frac{(1-f_{*1D}) \cdot p_d}{1-f_{*1}},$$

$$j_1 = \frac{j_a}{j_b}, \text{ and } j_2 = \frac{j_c}{j_d}.$$

Thus, f_{1*d} and f_{*1d} can be calculated as follows

$$f_{1*d} = \frac{t_1 \cdot f_{1*D}}{1+(t_1-1)\cdot f_{1*D}},$$

$$f_{*1d} = \frac{t_2 \cdot f_{*1D}}{1+(t_2-1)\cdot f_{*1D}}.$$

We can conclude that

$$\begin{aligned}
f_{11d} &= (1+K)\cdot f_{1*d}\cdot f_{*1d}\\
f_{10d} &= f_{1*d}-(1+K)\cdot f_{1*d}\cdot f_{*1d}\\
f_{01d} &= f_{*1d}-(1+K)\cdot f_{1*d}\cdot f_{*1d}\\
f_{00d} &= 1-f_{1*d}-f_{*1d}+(1+K)\cdot f_{1*d}\cdot f_{*1d}.
\end{aligned}$$

Thus, $p_{11d}, p_{10d}, p_{00d}$ and p_{01d} can be calculated as

$$\begin{aligned}
p_{11d} = &\frac{(p_{1*}f_{1*}+D_{X1,M1})(p_{*1}f_{*1}+D_{X2,M2})f_{11d}}{f_{1*}f_{*1}}+\\
&\frac{[p_{1*}(1-f_{1*})+D_{X1,M1}](p_{*1}f_{*1}+D_{X2,M2})f_{01d}}{(1-f_{1*})f_{*1}}+\\
&\frac{(p_{1*}f_{1*}+D_{X1,M1})[p_{*1}(1-f_{*1})+D_{X2,M2}]f_{10d}}{f_{1*}(1-f_{*1})}+\\
&\frac{[p_{1*}(1-f_{1*})+D_{X1,M1}][p_{*1}(1-f_{*1})+D_{X2,M2}]f_{00d}}{(1-f_{1*})(1-f_{*1})}
\end{aligned},$$

$$\begin{aligned}
p_{00d} = &\frac{[(1-p_{1*})f_{1*}+D_{X1,M1}][(1-p_{*1})f_{*1}+D_{X2,M2}]f_{11d}}{f_{1*}f_{*1}}+\\
&\frac{[(1-p_{1*})(1-f_{1*})+D_{X1,M1}][(1-p_{*1})f_{*1}+D_{X2,M2}]f_{01d}}{(1-f_{1*})f_{*1}}+\\
&\frac{[(1-p_{1*})f_{1*}+D_{X1,M1}][(1-p_{*1})(1-f_{*1})+D_{X2,M2}]f_{10d}}{f_{1*}(1-f_{*1})}+\\
&\frac{[(1-p_{1*})(1-f_{1*})+D_{X1,M1}][(1-p_{*1})(1-f_{*1})+D_{X2,M2}]f_{00d}}{(1-f_{1*})(1-f_{*1})}
\end{aligned},$$

$$p_{10d}=\frac{(p_{1*}f_{1*}+D_{X1,M1})[(1-p_{*1})f_{*1}+D_{X2,M2}]f_{11d}}{f_{1*}f_{*1}}+\frac{[p_{1*}(1-f_{1*})+D_{X1,M1}][(1-p_{*1})f_{*1}+D_{X2,M2}]f_{01d}}{(1-f_{1*})f_{*1}}+\frac{(p_{1*}f_{1*}+D_{X1,M1})[(1-p_{*1})(1-f_{*1})+D_{X2,M2}]f_{10d}}{f_{1*}(1-f_{*1})}+\frac{[p_{1*}(1-f_{1*})+D_{X1,M1}][(1-p_{*1})(1-f_{*1})+D_{X2,M2}]f_{00d}}{(1-f_{1*})(1-f_{*1})},$$

$$p_{01d}=\frac{[(1-p_{1*})f_{1*}+D_{X1,M1}](p_{*1}f_{*1}+D_{X2,M2})f_{11d}}{f_{1*}f_{*1}}+\frac{[(1-p_{1*})(1-f_{1*})+D_{X1,M1}](p_{*1}f_{*1}+D_{X2,M2})f_{01d}}{(1-f_{1*})f_{*1}}+\frac{[(1-p_{1*})f_{1*}+D_{X1,M1}][p_{*1}(1-f_{*1})+D_{X2,M2}]f_{10d}}{f_{1*}(1-f_{*1})}+$$

and $$\frac{[(1-p_{1*})(1-f_{1*})+D_{X1,M1}][p_{*1}(1-f_{*1})+D_{X2,M2}]f_{00d}}{(1-f_{1*})(1-f_{*1})}.$$

2. The relationship between D, Lewontin's D', and $D_{\max}$

In the main body of the literature, LD between the gene makers and disease genes is presented in D, so we can assume that $D_{X1,M1}$ be the linkage disequilibrium value of M_1 and X_1 and $D_{X2,M2}$ be the linkage disequilibrium value of M_2 and X_2.

When $D_{X1,M1}>0$, and $D_{X2,M2}>0$, we can assume that

$$D_{1\max}=\min\{f_{1*}\cdot(1-p_{1*}),\ p_{1*}\cdot(1-f_{1*})\},$$

$$D_{2\max}=\min\{f_{*1}\cdot(1-p_{*1}),\ p_{*1}\cdot(1-f_{*1})\},$$

$$D_1'=D_{X1,M1}/D_{1\max},$$

$$D_2'=D_{X2,M2}/D_{2\max}.$$

If $D_{X1,M1}=D_{1\max}$, $D_1'=1$, X_1 and M_1 are assumed as completely linked. If $D_{X1,M1}\neq D_{1\max}$, $0<D_1'<1$, X_1 and M_1 are assumed as incompletely linked. In the same way, the situation of X_2 and M_2 can be obtained.

3. The expectation of estimated effect size $E(\hat{\beta})$

From the expectation definition and the nature of covariance, we can get

$$
\begin{aligned}
&Cov(\hat{p}_{1*D}, \hat{p}_{*1D}) \\
&= E\{[\hat{p}_{11D} + \hat{p}_{10D} - E(\hat{p}_{1*D})][\hat{p}_{11D} + \hat{p}_{21D} - E(\hat{p}_{*1D})]\} \\
&= E\{[\hat{p}_{11D} + \hat{p}_{10D} - p_{1*D})][\hat{p}_{11D} + \hat{p}_{01D} - p_{*1D}]\} \\
&= E[(\hat{p}_{11D} + \hat{p}_{10D})(\hat{p}_{11D} + \hat{p}_{01D})] - p_{1*D} \cdot p_{*1D} \\
&= E(\hat{p}_{11D}{}^2 + \hat{p}_{10D} \cdot \hat{p}_{11D} + \hat{p}_{11D}\hat{p}_{01D} + \hat{p}_{10D}\hat{p}_{01D}) - p_{1*D} \cdot p_{*1D}
\end{aligned}
$$

.

Because of the equations below,

$$E(\hat{p}_{11D}{}^2) = Var(\hat{p}_{11D}) + E^2(\hat{p}_{11D}) = \frac{p_{11D}(1-p_{11D})}{n_D} + p_{11D}{}^2,$$

$$E(\hat{p}_{10D} \cdot \hat{p}_{11D}) = Cov(\hat{p}_{10D}, \hat{p}_{11D}) + E(\hat{p}_{10D})E(\hat{p}_{11D}) = -\frac{p_{10D} \cdot p_{11D}}{n_D} + p_{10D} \cdot p_{11D},$$

$$E(\hat{p}_{11D} \cdot \hat{p}_{01D}) = Cov(\hat{p}_{11D}, \hat{p}_{01D}) + E(\hat{p}_{11D})E(\hat{p}_{01D}) = -\frac{p_{11D} \cdot p_{01D}}{n_D} + p_{11D} \cdot p_{01D},$$

and

$$E(\hat{p}_{10D} \cdot \hat{p}_{01D}) = Cov(\hat{p}_{10D}, \hat{p}_{01D}) + E(\hat{p}_{10D})E(\hat{p}_{01D}) = -\frac{p_{10D} \cdot p_{01D}}{n_D} + p_{10D} \cdot p_{01D},$$

we obtain

$$
\begin{aligned}
&Cov(\hat{p}_{1*D}, \hat{p}_{*1D}) \\
&= E(\hat{p}_{11D}{}^2 + \hat{p}_{10D} \cdot \hat{p}_{11D} + \hat{p}_{11D} \cdot \hat{p}_{01D} + \hat{p}_{10D} \cdot \hat{p}_{01D}) - p_{1*D} \cdot p_{*1D} \\
&= \frac{p_{11D} \cdot (1-p_{11D})}{N_D} + p_{11D}{}^2 + (-\frac{p_{10D} \cdot p_{11D}}{N_D} + p_{10D} \cdot p_{11D}) + \\
&(-\frac{p_{11D} \cdot p_{01D}}{N_D} + p_{11D} \cdot p_{01D}) + (-\frac{p_{10D} \cdot p_{01D}}{N_D} + p_{10D} \cdot p_{01D}) - p_{1*D} \cdot p_{*1D} \\
&= \frac{p_{11D} \cdot (1-p_{11D}) - p_{10D} \cdot p_{11D} - p_{11D} \cdot p_{01D} - p_{10D} \cdot p_{01D}}{N_D} \\
&= \frac{p_{11D} \cdot (1-p_{11D} - p_{10D} - p_{01D}) - p_{10D} \cdot p_{01D}}{N_D} \\
&= \frac{p_{11D} \cdot p_{00D} - p_{10D} \cdot p_{01D}}{N_D}
\end{aligned}
$$

.

Due to
$$\begin{aligned} & p_{11D} - p_{1*D} \cdot p_{*1D} \\ &= p_{11D} - (p_{11D}{}^2 + p_{11D} \cdot p_{01D} + p_{10D} \cdot p_{11D} + p_{10D} \cdot p_{01D}) \\ &= p_{11D} \cdot (1 - p_{11D} - p_{01D} - p_{10D}) - p_{10D} \cdot p_{01D} \\ &= p_{11D} \cdot p_{00D} - p_{10D} \cdot p_{01D} \end{aligned},$$

we have $Cov(\hat{p}_{1*D}, \hat{p}_{*1D}) = \dfrac{p_{11D} - p_{1*D} \cdot p_{*1D}}{N_D}$.

In the same way, $Cov(\hat{p}_{1*d}, \hat{p}_{*1d})$ can be obtained as

$$Cov(\hat{p}_{1*d}, \hat{p}_{*1d}) = \frac{p_{11d} - p_{1*d} \cdot p_{*1d}}{N_d}.$$

From the aforementioned calculation process of $Cov(\hat{p}_{1*D}, \hat{p}_{*1D})$ and the calculation formula of $E(\hat{\beta}_D)$ in the main body of the literature, $E(\hat{\beta}_D)$ can be calculated as

$$\begin{aligned} E(\hat{\beta}_D) &= E(\hat{p}_{11D}) - E(\hat{p}_{1*D} \cdot \hat{p}_{*1D}) \\ &= p_{11D} - [E(\hat{p}_{1*D}) \cdot E(\hat{p}_{*1D}) + Cov(\hat{p}_{1*D}, \hat{p}_{*1D})] \\ &= p_{11D} - [p_{1*D} \cdot p_{*1D} + Cov(\hat{p}_{1*D}, \hat{p}_{*1D})] \\ &= p_{11D} \cdot p_{00D} - p_{10D} \cdot p_{01D} - Cov(\hat{p}_{1*D}, \hat{p}_{*1D}). \end{aligned}$$

In the same way, $E(\hat{\beta}_d)$ can be calculated as

$$E(\hat{\beta}_d) = p_{11d} \cdot p_{00d} - p_{10d} \cdot p_{01d} - Cov(\hat{p}_{1*d}, \hat{p}_{*1d}).$$

As both the sample size N_D and N_d are large enough, the above mentioned covariance is ignorable, we can get

$$E(\hat{\beta}_D) = p_{11D} \cdot p_{00D} - p_{10D} \cdot p_{01D}, \text{ and } E(\hat{\beta}_d) = p_{11d} \cdot p_{00d} - p_{10d} \cdot p_{01d}.$$

We therefore calculate the expectation of effect size as of below

$$E(\hat{\beta}) = (p_{11D} p_{00D} - p_{10D} p_{01D}) - (p_{11d} p_{00d} - p_{10d} p_{01d}).$$

4. Details of the statistical test and power calculation

In the main body of the literature, we propose the statistic as below

$$S = \frac{\hat{\beta}^2}{Var(\hat{\beta})},$$

when the distribution of effect size can be approximated in a normal distribution. We gave the null hypothesis (H_0) $\beta = 0$ that there is no interaction between X_1 and X_2 and the alternative hypothesis (H_1) $\beta \neq 0$, that is, there exists an interaction between X_1 and X_2. Under the null hypothesis, the statistic S follows a central chi-square distribution with a degree of freedom 1. We can conduct a statistical test $Test_1$ for statistic S. Let $Power_1$ be the statistical power of $Test_1$, which can be obtained under given threshold and degree of freedom using the *pchisq function* of R software (version 4.0.3, https://www.r-project.org/).

To compare the power of interaction identification with that of regular association test, we calculated the power of association test for the marker gene M_1 in the same circumstances.

Similar to the definition of f_{1*D}, f_{*1D}, and f_{11D}, f_{0*D}, f_{*0D}, and f_{00D} is frequency of chromosomes in case samples that carrying X_1, X_2, and both of them, respectively. The notation '*' indicates any allele of '0' or '1' at the current site, i.e. $f_{0*D} = f_{01D} + f_{00D}$.

Given the relative risks of X_1 and X_2 as R_{1*} and R_{*1}, the aforementioned frequencies in case samples are $f_{0*D} = \frac{R_{X1} f_{0*}}{R_{X1} f_{0*} + (1 - f_{0*})}$, $f_{*0D} = \frac{R_{X2} f_{*0}}{R_{X2} f_{*0} + (1 - f_{*0})}$, and $f_{00D}=(1+K) \cdot f_{0*D} f_{*0D}$, respectively. It is clear that the interaction coefficient K indicates

the extra LD between X_1 and X_2 due to their non-additive (multiplicative) risk. Similarly, we can obtain the frequencies f_{0*d} , f_{*0d} , and f_{00d} for the chromosomes in control samples.

In single-site genetic association studies, the odd ratio of gene frequency between the disease population and the general population is defined as

$$\phi = \frac{f_{1*D} / f_{0*D}}{f_{1*d} / f_{0*d}} = \frac{f_{1*D} \cdot f_{0*d}}{f_{0*D} \cdot f_{1*d}} .$$

The mathematical expectation of the odd ratio of gene frequency between the disease population and the general population is defined as

$$E(\hat{\phi}) = \frac{f_{1*D} \cdot f_{0*d}}{f_{0*D} \cdot f_{1*d}} .$$

The estimated variance of the odd ratio of gene frequency between the disease population and the general population is difficult to calculate, so we calculate the estimated variance of the odd ratio logarithm as

$$Var[\log(\hat{\phi})] \approx \frac{1}{N_D \cdot f_{1*D}} + \frac{1}{N_D \cdot f_{0*D}} + \frac{1}{N_d \cdot f_{1*d}} + \frac{1}{N_d \cdot f_{0*d}} .$$

We can compose a statistic

$$T = \frac{[\log(\hat{\phi})]^2}{Var[\log(\hat{\phi})]} .$$

Under the null hypothesis that the status of the gene site has nothing to do with the individual's disease status, the distribution of T will follow a central chi-square distribution with one degree of freedom. Under the alternative hypothesis that the status of the gene site is associated with the individual's disease status, the distribution of T will follow chi-square distribution with one degree of freedom. We can conduct a statistical

test $Test_2$ for statistic T. Let $Power_2$ be the statistical power of $Test_2$, which can be obtained under given threshold and degree of freedom using the *pchisq function* of R software (version 4.0.3, https://www.r-project.org/).
